# Supplementary material for: Harmonization of ICF Body Structures and ICD-11 Anatomic Detail: One foundation for multiple classifications
Source: PLoS One. 2023 Jul 27;18(7):e0280106. doi: 10.1371/journal.pone.0280106 (PMC10374130; doi:10.1371/journal.pone.0280106)
Supplement: Attachment — Submitted filename: Response to PLOS Reviewers.docx [file pone.0280106.s001.docx]

Response to Reviews’ Comments

We thank the reviewers for their comments and the opportunity to improve our manuscript.

**Reviewer #1: It's always a useful step to begin creating such links across classification systems, and this appears to be a responsible, exploratory effort to begin building those bridges of equivalence and similarity where existing terminologies and hierarchies permit.**

We thank the reviewer’s positive comment.

**However, this represents an admittedly small subset (434 relations) of all the complex and dynamic relationships that exist between the FIC terminologies. ICF has 1400 categories, and ICD-11 has 17,000 unique codes and more than 120,000 codable terms. If we consider the varieties of co-morbid conditions where multiple anatomical, physiological, and interventional factors may interact, our models will require both broader and more precise classifications. It's not clear to me what clinical advantage is gained by mapping 434 relations, in the midst of so many other un-mapped relations. It would be helpful in the Abstract to specify what specific scientific or clinical benefits can be expected from this improved cross-classification mapping.**

The reviewer is correct to point out the need to situate the reported mapping results in the larger context of the ICD-11 and ICF classifications. In fact the anatomical mappings reported in this paper represent a case study in the larger effort to harmonize the concepts in ICD-11, ICF, and ICHI. In the abstract we added the following to clarify the context of the work:

Recently, the three classifications have been incorporated into a single *WHO-FIC* *Foundation* that serves as the repository of all concepts in the classifications. Each classification serves a specific classification need. However, they share some common concepts that are present, in different forms, in two or all of them. For the WHO-FIC Foundation to be a logically consistent repository without duplicates, these common concepts must be reconciled.

In the Introduction section, we elaborated on the point as follows:

The most notable recent enhancement to the three classifications is that, following the development of a new architecture for ICD-11 (5), all of them have been incorporated into a single WHO-FIC Foundation that allows both ICF and ICHI to be generated as linearizations of the Foundation (6) which is an integrated semantic network consisting of all of the knowledge related to the concepts in the three classifications. Formalized in the Web Ontology Language (OWL), the Foundation organizes concepts in polyhierarchies that have no residual terms. The concepts are structured according to a WHO-FIC Content Model (7) that specifies the necessary attributes, such as titles and definitions, of a concept; its lexical properties, such as synonyms and inclusions; and the logical organization of the hierarchies, such as the allowed and required post-coordination axes. Different classifications, such as ICF, ICHI, and versions of ICD-11 designed for different use cases, can be generated automatically as different linearizations according to their specifications in the Foundation. The WHO-FIC Foundation promises to be a modern terminology system that is both backward compatible with the traditional requirements of statistical classifications and also prepared for linking and mapping to other terminologies and ontologies. Currently in this combined representation, ICD-11 and ICF concepts are represented separately, while ICHI, because of its recent provenance, references some ICD-11 and ICF concepts.

For the WHO-FIC Foundation to realize its promise, it needs to reorganize the ICD-11, ICHI, and ICF concepts so that they form a logically consistent and semantically integrated and linked whole.

**I'm also a bit concerned about the accepted constraints implicit in the harmonization initiative, which prioritize compliance "with the WHO approach to classification modeling and maintenance." This risks constraining future terminologies and relationships within pre-existing frameworks which may simply be, or become, outdated or too crude to represent emerging complexities of scientific discoveries (e.g., chemotherapy vs. bioelectric field therapeutics). The "least common denominator" of harmony may end up giving us classification models that are less usefully precise.**

In the text, we clarified that the “WHO approach to classification modeling and maintenance” is NOT that of the traditional statistical classifications. The new terminological architecture, consisting of a unified Foundation and multiple linearizations for different classifications (and different versions of classifications), is the current WHO approach to classification modeling and maintenance.

**Finally, I think we need to consider the growing relevance of semantic web data science and graph knowledge-base technologies as relevant platforms for making the vast scope of medical classifications and individual patient informatics more dynamically evolvable, make research more precisely and rapidly searchable, and make health informatics more interoperable. WHO's operational conservatism in classification may be locking the world of clinical research and services into obsolescent relational database frameworks of classification that will increasingly not mirror the dynamism and complex interdependencies of human health, Social Determinants of Health, clinical sciences and technological innovation.**

We agree with the reviewer on the need to be innovative. Given that ICD-11 was approved by the World Health Assembly only three years ago, we need to disseminate information about its new architecture better. The WHO-FIC Foundation, which is relevant to but not the focus of this paper, and from which the classifications are derived, is designed for the tasks described by the reviewer. As the excerpted text shown earlier, the WHO-FIC Foundation is formalized in the Web Ontology Language (OWL). The new post-coordination capabilities of ICD-11 and ICHI are based on description logic. The curation platform for the WHO-FIC Foundation is in fact a customized version of the industry standard OWL editor WebProtege. Groups in the WHO-FIC community are actively pursuing possible integration or linkage with the Mondo Disease Ontology and the Human Phenotype Ontology.

**Reviewer #2: The authors describe the three WHO classification systems (ICD-11, ICF, ICHI) and their different usage. Developed a user-friendly interface for coders to map the ICD-11 with ICF. Elaborated on the detail differen[ces] between ICD-11 and ICF in anatomy entities.**

**The article needs to be revised majorly for publication:**

**1. Describe the usage of this harmonization? Where and under what situation will the scientists or healthcare workers/administrators use this?**

As described in the response to Reviewer #1, the goal of the harmonization is to create a logically consistent WHO-FIC Foundation, a terminological system that forms the basis for different versions of WHO classifications. The benefits to scientists or healthcare workers and administrators will derive from these classifications and from the eventual use of the Foundation itself.

**2. Consider artificial intelligence methods. Use the experts’ results as training data for developing a model to reduce the experts’ efforts.**

We have added the reviewer’s suggestion as part of the future work. However, the size of the subset used in the present work is small and could not lead to usable models. Additional work is needed to make such methods possible.

**3. Map ICD-11, ICF and ICHI.**

As described in the response to Reviewer E1, the ICF/ICD-11 anatomy mapping is the part of a larger program of work that will indeed discover and map shared concepts in ICD-11, ICF, and ICHI.

**4. Supplement the Conclusion part.**

We added to the Conclusion part:

The mapping of anatomical concepts in the ICF body structures domains and ICD-11 Anatomy and Topography extension codes yields insights into how the two classifications treat the representation of anatomy structures differently. We present methods for reconciling the differences. The case study points to the way additional work harmonizing the concepts in ICD-11, ICF, and ICHI may be conducted.

**5. Describe the innovation of this research.**

We added to the Discussion part the following:

This work makes three-fold contributions: first, it presents a principled exemplar of an approach to design and conduct terminological mappings; second, it uncovers specific commonalities and discrepancies between the anatomical concepts in ICD-11 and ICF and proposes methods to harmonize them in the context of the WHO-FIC Foundation; third, it pioneered the tools and methods, and provides the first results of a larger program of work to map, integrate, and link concepts in the three core WHO-FIC classifications. Future work will investigate the relationships such as those between impairments of ICF body functions and ICD-11 signs and symptoms, between ICHI health interventions and ICD-11 external causes of injuries, and between ICF activities and participations and ICD-11 dimensions of dimensions of external causes, among others. Just as we developed the web-based software to assist subject matter experts performing the mappings, we will investigate additional automated methods to map related concepts, possibly leveraging previously validated mappings as training sets.
