# Supplementary material for: Harmonization of ICF Body Structures and ICD-11 Anatomic Detail: One foundation for multiple classifications
Source: PLoS One. 2023 Jul 27;18(7):e0280106. doi: 10.1371/journal.pone.0280106 (PMC10374130; doi:10.1371/journal.pone.0280106)
Supplement: Attachment — Submitted filename: 2nd Response to PLOS Reviewers Comments.docx [file pone.0280106.s002.docx]

**Reviewer #2: I deeply appreciate the authors' efforts in advancing the WHO-FIC through their work. It’s always useful to start from the coding step to try to manage and utilize healthcare data. But the author did not expound on the significance of their work. The WHO-FIC Foundation initially comprised the International Classification of Diseases (ICD), International Classification of Functioning, Disability and Health (ICF), and International Classification of Health Interventions (ICHI), which were designed to categorize distinct domains of health information. From a statistical and data analysis perspective, it is unclear how their work will be used in the future. In terms of medical terminology, pairing ICD and ICF appears to conflict with the primary aim of separating different fields of classification. It is worth noting that the citation does not indicate that the WHO-FIC Foundation intends to amalgamate the three coding systems.**

>> *Response*

*ICD, ICF, and ICHI, while each focusing on its specific health and health care domains, are designed to complement each other, not artificially separated. There are already closely related concepts in the classifications, for example, between ICF’s pain body functions and sensation and ICD-11’s pain symptoms. Especially with the introduction of extension codes in ICD-11, there are significant overlaps in the coverage of the three classifications. With joint use of the classifications, collected data need to be consistent (e.g., ICD-11 codes with anatomical details and ICF body structure impairment). Consistent coding practices in joint use of the classifications will facilitate coordinated care and enhance data integration and analysis. In the revised paragraph in the Introduction, we make these points explicitly and cite a couple examples of the joint use of ICD and ICF.*

For the WHO-FIC Foundation to realize its promise, it needs to reorganize the ICD-11, ICHI, and ICF concepts so that they form a logically consistent and semantically integrated and linked whole. Even though they cover different needs, the classifications share common concepts that are currently present in different forms in the Foundation. ICF, for example, includes body functions such as “Sensation of pain” (b280) that are closely related to ICD-11’s pain symptoms. Furthermore, with the introduction of post-coordination (8), ICD-11 includes extension codes, such as anatomical entities and health devices, equipment, and supplies, that are not in past versions of ICD and that increase the possibility of overlaps among the classifications. Identifying, characterizing, and harmonizing all the possible shared concepts is a huge and complex task. It is the aim of a current WHO effort to harmonize the shared concepts among the reference classifications of the family so that duplicates are removed and related concepts are organized in consistent hierarchies. Such harmonized concepts across classifications will facilitate the joint use of the classifications, for example in documenting disability characteristics of children in early intervention (9) or determining functioning and disability profiles of chronic stroke patients (10). ICD-11 codes post-coordinated with anatomical details and ICF-coded data on body structure impairments can be made interoperable just as ICHI health intervention codes already use ICF body functions and activities and participation codes as targets. Such consistent coding practices will facilitate coordinated care and enhance data integration and analysis. These benefits to users of the classifications and of the Foundation cannot be realized until this harmonization is completed.

(8) Mabon K, Steinum O, Chute CG. Postcoordination of codes in ICD-11. BMC Med Inform Decis Mak. 2022;21(Suppl 6):379.

(9) Martins EF, de Sousa PH, Barbosa PH, de Menezes LT, Costa AS. A Brazilian experience to describe functioning and disability profiles provided by combined use of ICD and ICF in chronic stroke patients at home-care. Disabil Rehabil. 2011;33(21-22):2064-74.

(10) Simeonsson RJ, Scarborough AA, Hebbeler KM. ICF and ICD codes provide a standard language of disability in young children. J Clin Epidemiol. 2006;59(4):365-73.

*We are surprised that the reviewer said "***the citation does not indicate that the WHO-FIC Foundation intends to amalgamate the three coding systems***.” While the integration of the three classification in a single WHO-FIC Foundation is work in progress and is not yet described in journal publications, the Foundation reference (#6 in the reviewed submission) points to a web page that clearly shows that the three classifications are in the Foundation. If the reviewer’s point is that the three classifications will continue to exist as separate classifications for coding, that is true, but misses the point that they should be generated as linearizations from the single WHO-FIC Foundation where all of the classification concepts are amalgamated and made consistent.*

**>> Reviewer’s comment**

**Based on my understanding, the involvement of human interaction in the development of natural language processing (NLP) methods for healthcare is critical but can be both time and cost-intensive. If the authors make their data publicly available, it could prove to be a valuable resource for training NLP methods. In this regard, I would encourage the authors to present their matching results in such a way that they could serve as golden standards for algorithm training, particularly with regard to entity recognition.**

*>> Response*

*As stated in the reviewed manuscript "*The consolidated maps are made available in SSOM format (12) at<https://github.com/whoficitc/harmonization/blob/main/ICF-ICD-anatomy-v1.tsv>.*” SSOM format is a simple tabular structure that is easy for anyone who wants to re-use the data to reformat for their purpose.*
